# A prospective observational study regarding host-derived immunological parameters around zirconia implants in comparison to natural teeth following flap surgery

**DOI:** 10.1038/s41598-025-10902-5

**Published:** 2025-07-17

**Authors:** Kristian Kniha, Lothar Rink, Marius Heitzer, Stephan Christian Möhlhenrich, Marie Sophie Katz, Anna Bock, Frank Hölzle, Ali Modabber

**Affiliations:** 1 Department of Oral and Cranio-Maxillofacial Surgery, University Hospital RWTH Aachen, Pauwelsstraße 30, Aachen, Germany; 2 Private clinic for oral surgery, Dres. Kniha, Rosental 6, 80331 Munich, Germany; 3 Institute of Immunology, University Hospital RWTH Aachen, Pauwelstraße 30, Aachen, Germany; 4 Department of Orthodontics, University of Witten/Herdecke, Alfred-Herrhausen Str. 45, 58455 Witten, Germany; 5 Department of Oral and Cranio-Maxillofacial, Pauwelsstraße 30, 52074 Aachen, Germany

**Keywords:** Dental implant, Ceramic, Zirconia, Fibula flap, Deep circumflex Iliac artery, Health care, Dentistry, Dental treatments, Dental implants

## Abstract

With the increasing use of dental implants in patients undergoing extensive mandibular reconstructions, it is crucial to understand how soft tissues react in different implantation contexts. The aim was to compare the behavior of the soft tissues surrounding zirconia implants to that of the soft tissues surrounding natural teeth in terms of cytokine levels in patients who had undergone various microvascular flap procedures for jaw reconstruction. Due to anatomical deviations after flap surgery, such as thick skin paddles, the possibility of fixed implant dentures in patients with bony flaps is rare. Therefore, these patients are often treated with removable dentures. In this prospective observational study ten patients with a total of six fibula flaps and four deep circumflex iliac artery (DCIA) flaps underwent reconstruction in the lower and upper jaws using vascularized bone flaps, and were treated with a total of 41 zirconia implants. The cytokine levels in the crevicular fluid were analyzed in terms of the interleukin-1 beta (IL-1b) and matrix metalloproteinase-8 (MMP-8) levels up to one-year follow-up. The implant survival and success rates were also investigated up to one year. No significant differences in IL-1b were found between natural teeth and ceramic implants. After six months, MMP-8 levels of the natural teeth of a patient treated with DCIA flaps were once significantly lower when compared to the ceramic implants inserted into fibula flaps (*p* = 0.001). The overall survival and success rates were 100 and 76.83%, respectively. For the fibula group, the survival and success rates were 100 and 72.55%, respectively, and for the DCIA group, they were 100 and 81.12%. Zirconia implants and natural teeth showed comparable cytokine levels in the crevicular fluid. Nevertheless, implant treatment with extensive microvascular jaw reconstructions affected the success rates in the present study.

## Introduction

In cases of significant jaw defects, microvascular bony flaps offer an efficient reconstruction treatment option with low graft resorption^1^. The iliac crest and fibula are well-established donor sites for upper- and lower-jaw reconstructions^2–4^. Nevertheless, different problems like bone resorption and soft tissue inflammation around titanium implants inserted in different bone grafts or in different microvascular flaps are described in the current literature^5–7^. This inflammatory reaction needs to be researched and avoided in the future.

Cytokines are signaling proteins that mediate and regulate immunity and inflammation^8^. Peri-implant immunological markers are crucial in assessing the health and stability of dental implants^8^. These markers help in detecting and understanding the immune response around dental implants, potentially indicating inflammation or peri-implantitis, which can lead to implant failure. Key-immunological markers in periimplant health are Interleukin-1 (IL-1) and Matrix Metalloproteinases (MMP-8)^9^. On the one hand elevated levels of IL-1, particularly IL-1β, are associated with inflammation and bone resorption around implants. On the other hand MMPs enzymes are involved in the breakdown of extracellular matrix components, important in tissue remodeling and inflammation. High IL-1 and MMP-8 parameters show a high degree of inflammation, as Interleukin-1 induces a local inflammatory reaction and salivary matrix metalloproteinase (MMP)-8 is currently considered to be one of the most promising biomarkers for early diagnosis of periodontitis^10^.

Immunological markers can be used to diagnose peri-implant diseases early, even before clinical signs become apparent. Monitoring these markers can help in assessing the effectiveness of treatment interventions^11^.

Due to the extensive long-term studies on the titanium implant material, it is currently regarded as the gold standard in dental implantology^12,13^. At the time that titanium dental implants were being developed, ceramic materials were also being tested as alternatives^14^.

Due to encouraging research findings, ceramics gained practical relevance in the early 2000s. As a result, ceramic—often referred to as zirconia—has become increasingly popular^15^ Zirconia has clearly prevailed over other ceramics, such as aluminum hydroxide ceramic, due to its excellent biomechanical properties^16–18^.

The current literature discusses a number of issues, including bone resorption and soft tissue inflammation surrounding titanium implants inserted into various microvascular flaps^7,19^. Despite the rarity of studies with difficult hard and soft tissue situations, zirconia implants already offer a dependable option for single-tooth gaps^20^.

The aim of the present study was to investigate the soft tissue behavior regarding cytokine levels around zirconia implants compared to natural teeth in patients who had been treated with different microvascular flaps for jaw reconstruction. The survival and success rates of the implants of the fibula and deep circumflex iliac artery (DCIA) flaps were also compared. The null hypothesis was tested under the assumption that zirconia implants as tooth replacement would not affect the IL-1b cytokine levels in the crevicular fluid.

## Materials and methods

Patients with reanastomized microvascular bony flaps were enrolled in the present prospective observational study. Patients were consecutively recruited from January 2016 to March 2021. The evaluation of this patient collective focuses on the sulcus fluid.

All patients who underwent dental implant therapy in fibula or DCIA flaps and who had follow-up examinations without experiencing any malignant relapses were included. The use of zirconia implants with fixed dentures was another requirement for inclusion. Patients with systemic disorders (e.g., uncontrolled diabetes), smoking habits, and radiotherapy after dental implant placement were excluded. The study protocol was examined and approved by the local medical faculty university’s ethical committee (Ethics Committee of the Faculty of Medicine of the RWTH Aachen University, Nr. 189/15). The study was conducted in accordance with the principles of the Declaration of Helsinki and the statement (STROBE) of strengthening the reporting of observational studies in epidemiology^21^.The Declaration of Helsinki’s principles and the statement enhancing the reporting of observational studies in epidemiology were followed during the study’s execution^21^. The study was registered at the clinical trails database DRKS (Bundesinstitut für Arzneimittel und Medizinprodukte; Bonn, Germany, Nr. DRKS00033774, pre-registration on 23.03.2024).

**Fig. 1:**
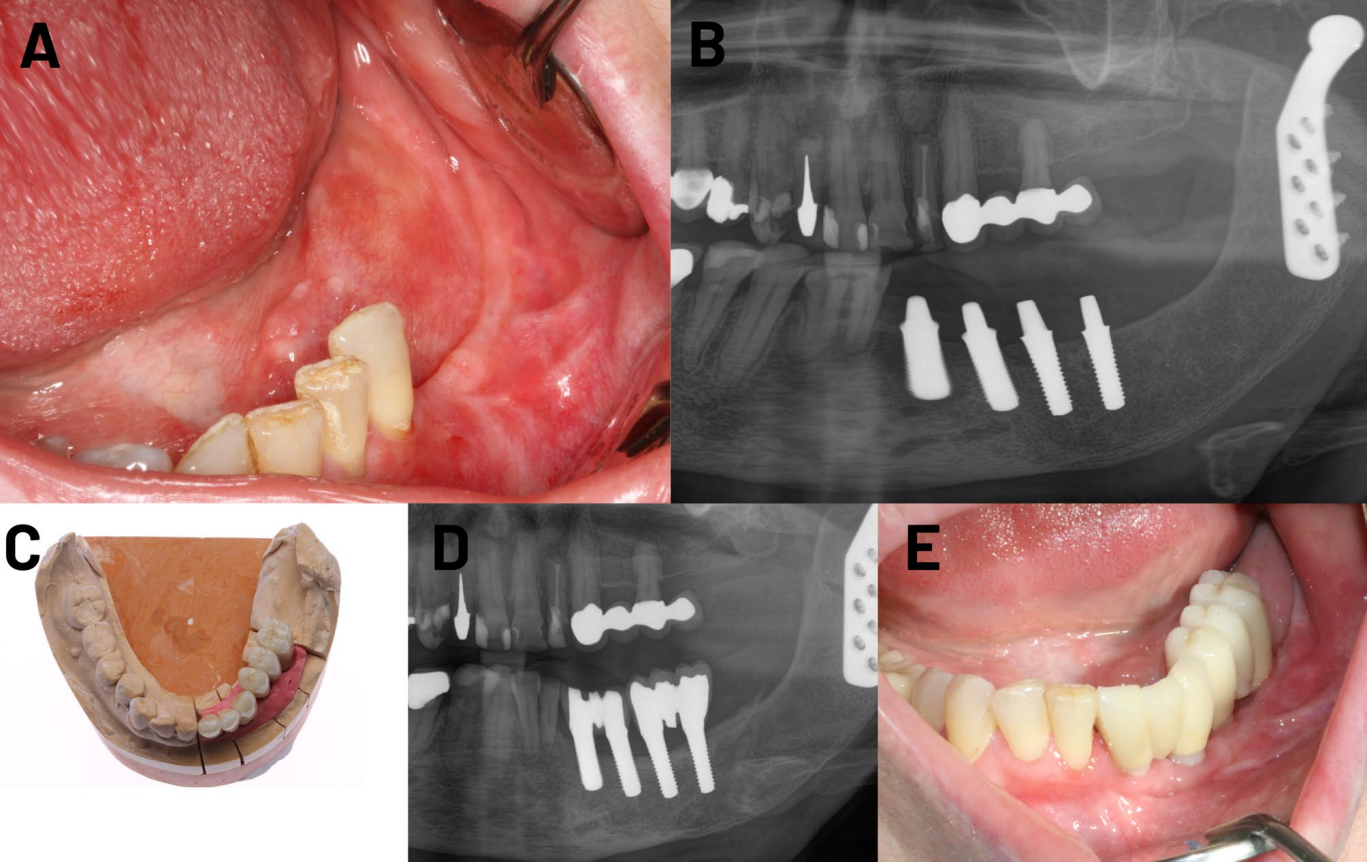
(**A**) This patient underwent a lower-jaw resection of the lift mandible due to osteomyelitis. The clinical picture shows the missing teeth and the reconstruction in the lower jaw using vascularized fibula flap. (**B**) The X-ray shows the lower jaw after implant placement. The patient was treated with four zirconia implants. (**C** and** D**) After a 3-month healing period, the implants were loaded onto a fixed dental bridge. (**E**) Clinical picture after one-year follow-up with the final dental crowns.

The flap types depended primarily on the location of the jaw defect and the size of the defect. For example, for complete mandibular defects and maxillary defects, the fibula flap is mostly advantageous. On the other hand, the DCIA flap is advantageous for a unilateral mandibular reconstruction or a subtotal defect. Individual patient characteristics such as previous operations also play an important role. Nine to twelve months after reconstructive surgery using fibula or DCIA flaps, the osteosynthesis plates and screws were removed if the general and oncological circumstances permitted. The implant procedure was carried out after another 3 months. Fixed dentures were placed after 3 more months of implant integration, and the soft tissue condition was assessed after implant recovery (Fig. 1).

Additional soft tissue surgery, such as vestibuloplasty, was done when necessary and appropriate using a free gingival grafts from the palate. The size of the graft depended on the recipient site. At the recipient site an epiperiosteal flap plasty with apical sliding flap was performed .The gingival graft was harvested from the palate with a scalpel at a thickness of approximately 1.5 mm and sutured to the periosteum of the recipient site. Patients were instructed to use a standard mouthwash 3 times a day and stitches were removed after 10 days.

### Immunological analysis

**Fig. 2:**
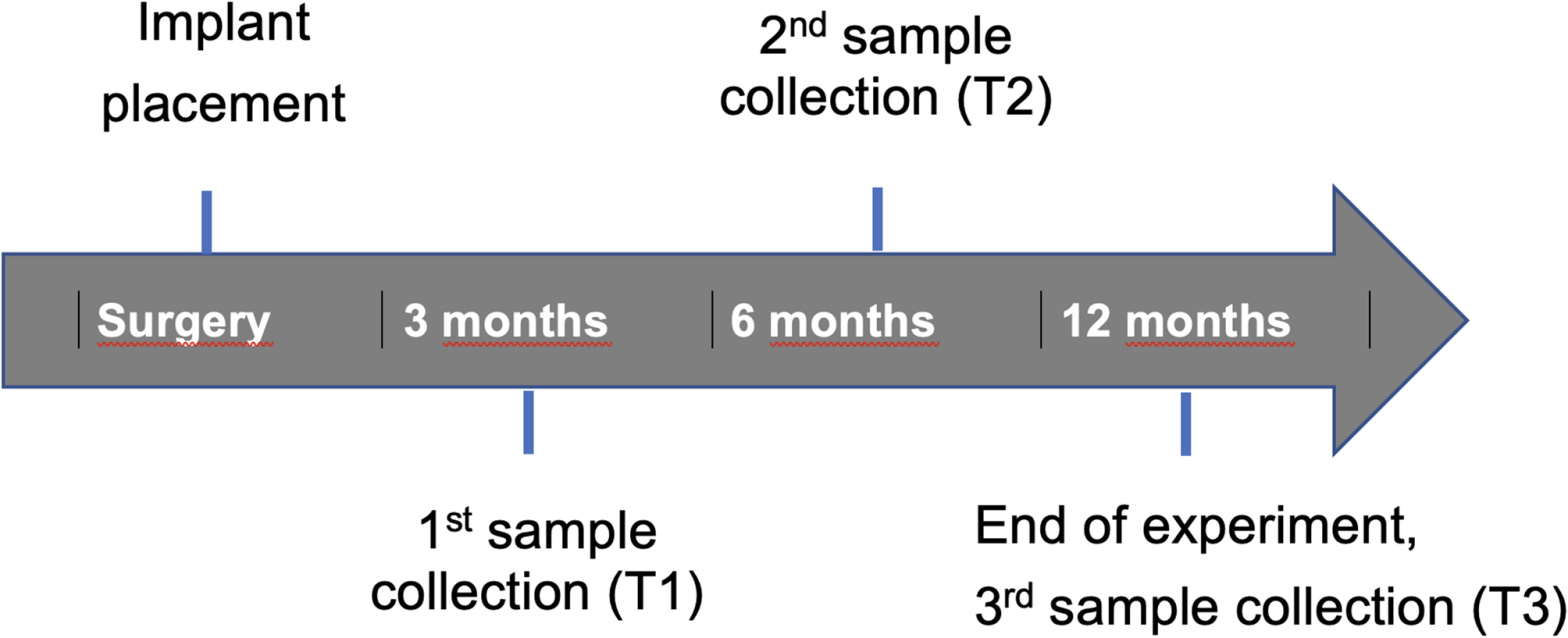
Clinical procedure and sample collection.

This protocol has been published in a previous study evaluating matrix metalloproteinase-8 (MMP-8) and interleukin-1 beta (IL-1b) levels^22^. Immunological parameters were measured at three sessions around each zirconia implant and the contralateral natural tooth: first after placement of the final crowns (session 1, 3 months after insertion), second after 6 months (session 2), and third after 12 months (session 3) (Fig. 2).

The deepest probing or pocket depth surrounding each implant or contralateral tooth that was the deepest side at the baseline measurements was sampled using sterile paper points (Paper points 29 mm; ISO 25; Taper.02; VDW; Munich, Germany). Each implant was measured and evaluated. For each implant, a tooth was measured, leading to the same number of samples. The samples were obtained from the same unit side in each session. The tips with a defined amount of fluid were stored in one tube filled with saline (350 µl phosphate-buffered saline; Sigma-Aldrich; St. Louis, Missouri, USA) + serum (10% fetal calf serum PAA; Eppendorf tubes; 1.5 ml; VWR International; Langenfeld, Germany) at -80 °C.

For analysis, each sample was thawed only once. ELISA was used to detect cytokines according to the manufacturer’s recommendations. MMP-8 and IL-1b were measured using antibody pairs (antibody pairs; OptEIA; BD Pharmingen; Heidelberg, Germany).

The ELISA was quantified on an reader (Ultra384 ELISA reader; Tecan; Männedorf, Switzerland). The storage buffer’s dilution factor (88.5) and the specified recovery coefficient were multiplied to determine the final cytokine concentrations. After the sampling sessions, the fibula flaps were compared to the DCIA flaps.

### Implant success

The success rate according to Albrektsson et al. was used^23^. Albrektsson et al. (1986) postulated a combination of anamnestic data, clinical examination results and radiological findings for the success rate of implants: no pain or discomfort, immobility, absence of radiolucency, and bone resorption of less than 0.2 mm per year from the timepoint of implant loading. The modified sulcus bleeding index was measured on four surfaces around the implants and was scored as follows: 0 = no bleeding; 1 = isolated bleeding; 2 = confluent linear bleeding; and 3 = severe bleeding. Another important parameter, pocket depth, was measured at four points around each implant and the corresponding contralateral tooth. An average value was then calculated from the 4 measurements. An experienced clinician recorded all the measurements at session 3 using a probe with a standardized probing force of 0.2 N. Radiographic pictures were taken immediately after implant surgery and after three, 12, and 18 months. To evaluate bone resorption for implant success, digital panoramic radiographs (Sirona, Bensheim, Germany) were obtained using a previously published measuring method^24^.

### Statistical analysis

Post hoc power analyses were performed with a software (G*Power 3; Version 3.1.9.2; Düsseldorf, Germany, Faul et al.^25,26^). The analysis of variance for repeated measures with a post hoc test was used as an indication for power. It was hypothesized that zirconia implants and natural teeth present comparable cytokine levels in the crevicular fluid. Analyses determined the power of 1.00 (primary study aim) based on the sample size 41 using an effect size of 0.67 ^27^ and an α of 0.05.

Analyses were performed using a software for Mac OS X (Prism 8; GraphPad; La Jolla, California, USA) running on Apple OS X. The analysis values were tested for normal distribution using the Kolmogorov–Smirnov normality test. Time point and tooth-implant type were parts of the model, along with the interaction between the two factors, to determine if the response was different over time for the three surface groups. Additionally, post hoc Tukey’s multiple comparison test was used to identify the differences between the means of the subgroups. Tukey’s post hoc test was used because we wanted to determine which specific group means are different after finding a significant overall effect. This includes the Multiple Comparisons, which allows for pairwise comparisons between all groups, controlling for the family-wise error rate.

Overall, Tukey’s test is a powerful tool for exploring differences in group means when you have already established significant differences with ANOVA. data includes time factors, implant/tooth type, and the interaction between these factors. A correlation analysis (Spearman r) was performed to evaluate a statistical link between the IL-1b and MMP 8 values and the clinical parameters. Any effect in the statistical model was assessed as significant if the corresponding p-value fell below the 5% margin.

## Results

Ten patients (5 males, 5 females, mean age of 51 years) with a total of 41 zirconia implants were included in the present one-year follow-up study (Table 1). Six patients were treated with a fibula flap, and 4 patients with a DCIA flap. For the underlying initial disease, the following occurred (with the frequency of occurrence): adenoid cystic carcinoma (*n* = 1), cancer of unknown primary syndrome (*n* = 1), trauma (*n* = 1), neurofibromatosis (*n* = 1), osteomyelitis (*n* = 2), and plaque epithelial cancer (*n* = 4). After benign tumor resection, primary bony reconstruction was performed. In cases of malignant tumor resection, secondary bony reconstructions were conducted if there was no malignant relapse after 12 months. All the DCIA flaps were myoosteo-, and all the fibula flaps were osteomyocutan reconstructions. All the implants were inserted in bony reconstructions that had not undergone radiotherapy. In the lower jaw, all the flaps that were used were DCIA flaps, and in the upper jaw, fibula flaps were used. Vestibuloplasty was done in two cases (1x DCIA flap and 1x fibula flap). As the implants were inserted after the flap surgery, all patients had a normal swallowing act again. After the implant restoration, all patients showed normal chewing function. Nevertheless, the patients needed a longer time to get used to the correct bite height. Normal hygiene of the implants was ensured in all cases and was carried out correctly. Therefore, all patients were instructed in oral hygiene before the operation. This included regular brushing with a toothbrush twice a day. In addition, dental floss and mouthwash (water) should be used regularly to remove plaque.

**Table 1:** Demographic table of all implant positions.

Implant position	17	16	15	14	13	12	11	21	22	23	24	25	26	27	
N		1		1	2		1	2	1	1	1		1		Total 41
N	2	4	2	4	3			1	1	4	4	2	3		
Implant position	47	46	45	44	43	42	41	31	32	33	34	35	36	37	

**Fig. 3:**
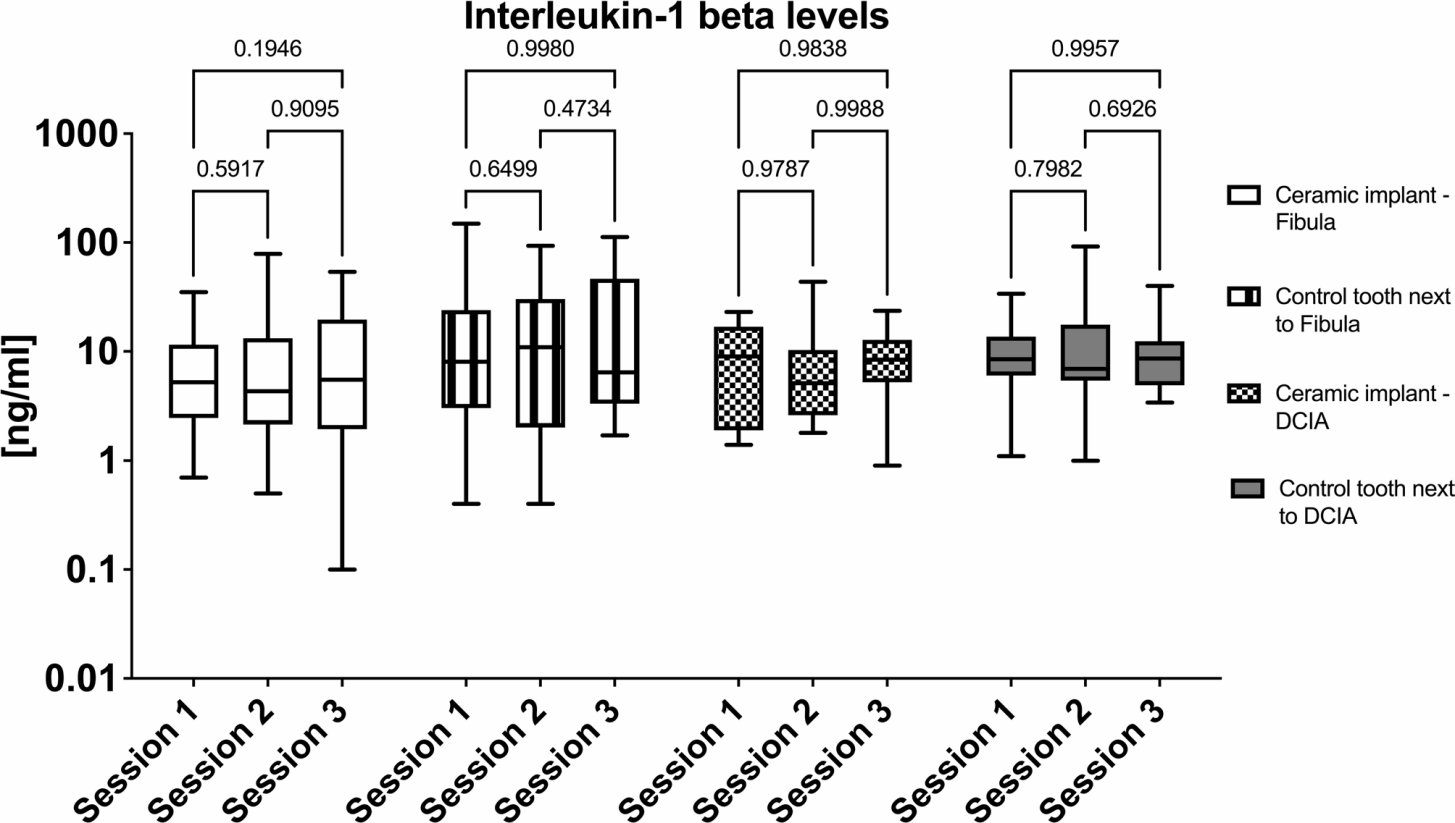
Statistical comparison of the interleukin-1b level. Sampling was performed first at session 1 after placement of the final crowns (3 months after implant insertion), second after 6 months (session 2), and third after 12 months (session 3). The comparison between the flaps and over time are presented.

Regarding the immunological analysis, fibula and DCIA flaps were compared, and ceramic implants were compared with natural teeth. Regarding the IL-1b levels, no significant differences were found between natural teeth and ceramic implants. Furthermore, no differences between the two flaps were recorded (Fig. 3, IL-1b levels).

**Fig. 4:**
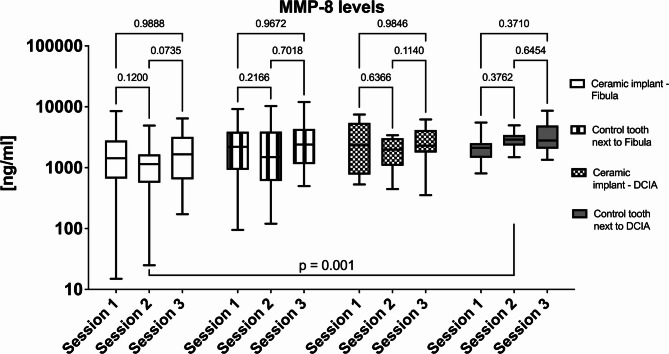
Comparison of MMP-8 levels. A significant difference was found between the groups at session 2 (ceramic implant fibula group versus contralateral tooth in the deep circumflex iliac artery groups; *p* = 0.001). The comparison between the flaps and over time are presented.

The MMP-8 levels were also investigated (Table 2, descriptive statistics ). At session 2, a significant difference was found between ceramic implants inserted into fibula flaps and natural teeth of patients that have been treated with DCIA flaps (Fig. 4, *p* = 0.001, MMP-8 levels). However, all the other comparisons showed no significant differences.

**Table 2:** Detailed immunological parameters and presentation of descriptive statistics.

	Human interleukin 1 ß					
	Fibula			DCIA		
Session	1	2	3	1	2	3
Zirconiaimplant						
N	29	29	29	11	11	11
Minimum	3.50	0.90	1.70	1.40	1.80	0.90
Maximum	19.00	79.00	79.00	23.00	44.00	24.00
Mean ± SD	9.30 ± 4.50	11.00 ± 17.00	13.00 ± 18.00	9.70 ± 8.10	9.00 ± 12.00	9.20 ± 6.10
Natural tooth						
N	29	29	29	11	11	11
Minimum	1.40	0.80	1.00	1.10	1.00	3.40
Maximum	26.00	93.00	93.00	34.00	92.00	40.00
Mean ± SD	12.00 ± 8.30	27.00 ± 29.00	28.00 ± 29.00	12.00 ± 11.00	18.00 ± 26.00	13.00 ± 13.00

	MMP-8					
	Fibula			DCIA		
Zirconiaimplant						
N	29	29	29	11	11	11
Minimum	15.00	25.00	25.00	530.00	450.00	350.00
Maximum	8500.00	4900.00	4900.00	7500.00	3400.00	6200.00
Mean ± SD	1900.00 ± 1800.00	1300.00 ± 1100.00	1300.00 ± 1100.00	2700.002400.00 ±	1900.00 ± 1000.00	2900.00 ± 1700.00
Natural tooth						
N	29	29	29	11	11	11
Minimum	95.00	120.00	120.00	810.00	1500.00	1300.00
Maximum	9200.00	10,000.00	10,000.00	5500.00	5000.00	8600.00
Mean ± SD	2900.00 ± 2800.00	2700.00 ± 2900.00	2500.00 ± 2500.00	2300.00 ± 1300.00	3000.00 ± 1100.00	3600.00 ± 2300.00

The survival and success rates of zirconia implants are presented in Table 3 (descriptive statistics). The overall survival rate was 100%, and the overall success rate was 76.83%. In the fibula group, the survival rate was 100%, and the success rate was 72.55%; in the DCIA flap group, they were 100% and 81.12%, respectively. The clinical parameters revealed a 2.5 mm (SD 1.5) pocket depth in the DCIA flaps around the implants and a 2.2 mm (SD 0.4) pocket depth around the natural control teeth. However, the pocket depth around the implants in the fibula flaps with 3.2 mm (SD 1.7) was higher than that of the natural control teeth with 1.9 mm (SD 0.5). The flaps had slightly higher sulcus bleeding index scores than the natural teeth, with no differences between the flaps. No complications such as crown- or implant chipping were evaluated.

**Table 3:** Survival and success rates of zirconia implant, mean and standard deviation values after 12-month follow-up of the pocket depths (PDs) and the sulcus bleeding indices (SBI) of zirconia implants and the contralateral natural teeth.

	DCIA		Fibula	
12 months	Survival rate %	Success rate %	Survival rate %	Success rate %
Implant	100	81.12	100.00	72.55
3 months	PD (mm) Mean (± SD)	SBI (0–3) Mean (± SD)	PD (mm) Mean (± SD)	SBI (0–3) Mean (± SD)
Implant	2.2(± 0.6)	0.2(± 0.6)	3.1(± 1.1)	0.2(± 0.8)
Tooth	1.6(± 0.9)	0.1(± 0.3)	1.6(± 0.6)	0.1(± 0.3)
6 months	PD (mm) Mean (± SD)	SBI (0–3) Mean (± SD)	PD (mm) Mean (± SD)	SBI (0–3) Mean (± SD)
Implant	2.4(± 0.9)	0.2(± 0.9)	2.6(± 0.9)	0.1(± 0.4)
Tooth	1.9(± 0.6)	0.0(± 0.0)	1.7(± 0.5)	0.1(± 0.1)
12 months	PD (mm) Mean (± SD)	SBI (0–3) Mean (± SD)	PD (mm) Mean (± SD)	SBI (0–3) Mean (± SD)
Implant	2.5(± 1.5)	0.6 (± 1.0)	3.2(± 1.7)	0.7 (± 1.1)
Tooth	2.2(± 0.4)	0.0 (± 0.0)	1.9(± 0.5)	0.1 (± 0.1)

A correlation analysis was performed to evaluate a statistical link between the IL-1b and MMP 8 values and the clinical parameters. The cytokine levels after twelve months were compared with the pocket probing depth and the bleeding on probing values. Only in one case, when comparing IL-1b levels and BOP values of ceramic implants in the fibula group, a significant correlation (Spearman *r* = 0.0058) was evaluated.

As qualitative observations of the cases, it can be concluded that the clinical parameters such as pocket probing depth and the bleeding on probing index showed changes in the observation period of twelve months and were visible during follow-up. These observations could not be linked to the cytokine values within one case. This aligns with the results, that the cytokine values showed no significant differences in either the test or the control group with natural teeth, except in one comparison.

## Discussion

Bony flaps that are treated with fixed protheses are rare. Most of the time, these patients are treated with removable dentures, as cleaning is very difficult with thick skin paddles. In these patients the anatomical deviations after flap surgery are very dramatic and show limited possibilities for fixes dentures (Fig. 1A and B). Not only did these patients received a fixed dental restoration, which represents a higher-quality restoration, but the patients also were treated with modern zirconia implants. This is a novelty in the treatment of this patient cohort.

Based on a previously published study design, we have chosen the examination dates three months after surgery, after six months and after twelve months^27^. After three months, the implants are prosthetically restored and thus represent a good start after successful implant integration. The one-year analysis is an important time for establishing the implant success and survival rate.

The material behavior and success rate of these implants are of great importance and could improve the treatment of our patients in the future. The success rate of zirconia implants in our study (overall success rate was 76.83%) was in the same range as that of titanium implants in the same cohort of patients. The success rate in this cohort of patients was reduced compared to healthy patients (Table 3). The short-term course of zirconia implants has been the subject of numerous one-year studies, all of which have shown encouraging survival and success rates^28,29^. Numerous studies have also looked at the midterm outcomes (between 2 and 5 years after implant insertion)^30–33^. As zirconia implants have been developed only recently, there has been limited long-term research on them. Thus, gathering initial long-term data is crucial. Due to the individual patient selection, data on implants inserted in bony flaps are scarce. These patients suffer greatly and, in many cases, cannot be adequately treated without implants. For this reason, a regulation in Germany covers implant costs through statutory health insurance. Hospital records and prior research were presented by Rogers et al. to identify consecutive maxillary resection cases related to head and neck cancer that occurred over a 27-year span, from January 1994 to November 2020. The clinical features, prosthetic rehabilitation, reconstruction, and survival were the main topics of the case note review^34^. The majority currently have free tissue reconstruction, and the use of primary zygomatic implants and early-loaded implant-supported fixed bridge reconstruction for oral rehabilitation are given more importance^34^.

Matrix metalloproteinases (MMP-8) and interleukin-1 (IL-1) are important immunological indicators of periimplant health^9^. We have chosen both parameters for this study, because interleukin-1 causes a local inflammatory response and salivary matrix metalloproteinase (MMP)-8 is currently regarded as one of the most promising biomarkers for early identification of periodontitis, high levels of IL-1 and MMP-8 characteristics indicate a high degree of inflammation^10^.

In the present one-year follow-up study, the peri-implant crevicular fluid of fibula and DCIA flaps treated with zirconia implants was assessed. The IL-1b levels showed no significant differences between the natural teeth and ceramic implants (Fig. 3). As such, the results of this experiment rejected the null hypothesis that zirconia implants as tooth replacement would not affect the IL-1b cytokine levels in the crevicular fluid. Furthermore, no differences between the two flaps were recorded. Clever et al. showed that the IL-1 levels varied significantly between the groups in their study (zirconia versus titanium and titanium versus tooth), with titanium implants having higher IL-1 levels^22^. A cross-sectional assessment showed that zirconia implants had significantly higher IL-1 levels than natural teeth^35^. There were no discernible differences between titanium and zirconia implants.

Kumar et al. assessed the MMP-8 levels in peri-implant crevicular fluid. At 1 and 3 months, the titanium abutments showed significantly higher MMP-8 levels and probing depths than the zirconia abutments^36^. In our study most of the comparisons showed no significant differences regarding MMP-8 levels (Fig. 4). Only the natural tooth in the DCIA group showed higher MMP-8 levels when compared ceramic implants inserted into fibula flaps. This fact is very interesting, as higher inflammation values and therefore higher MMP-8 levels would be expected around the implants. One possible cause could be that the oral flora around teeth and implants generally behave differently. MMP-8 assays are carried out on saliva or gingival fluid, and it has been demonstrated that patients with periodontitis have greater MMP-8 levels than healthy subjects do^37^. These levels have also been connected with certain clinical aspects of the disease and its severity. Furthermore, it has been shown that this enzyme is initially helpful in assessing the efficacy of doxycycline medication and periodontal therapy. The MMP-8 levels were compared in another study on titanium and zirconia healing caps^38^. The results showed a higher rate of restorative processes in the soft tissues around the titanium healing caps, which was likely connected to the MMP-8 levels found in the tissues.

A straightforward and reliable measure of the condition of the peri-implant tissues is bleeding on probing. The presence or degree of peri-implant mucositis can be associated by the degree of bleeding and the possibility of progression into peri-implantitis^39^. The average pocket depth in our study suggests an irritation-free periimplant soft tissue. The clinical parameters revealed a 2.5 mm pocket depth in the DCIA flaps around the implants and a 2.2 mm pocket depth around the natural control teeth (Table 2). However, the pocket depth around the implants in the fibula flaps with 3.2 mm was higher than that of the natural control teeth with 1.9 mm. Therefore, the slightly increasing bleeding on probing values could be an indication of a beginning mucositis. A close recall of the patients is recommended here.

Zirconia is highly biocompatible, reducing the risk of allergic reactions or adverse tissue responses^40^. Additionally, zirconia implants are often preferred for patients seeking optimal aesthetics, particularly in visible areas^41^. For patients needing a long-term solution, titanium may be favored due to its extensive research and proven outcomes^42^. Titanium implants can be used in a variety of clinical situations, including immediate loading protocols^43^.

It must be noted that the patients in the present study collectively differed from those examined in most studies by the extended operation undergone by them. Without major pre-operation, better anatomical prerequisites are usually given for dental implant placement. Studies have already assessed titanium implants inserted into different flaps. In Mertens et al.’s study, the vertical bone resorption rate was 6.79% for the patients in the DCIA group after a mean observation time of 6 months, 10.20% after 11 months, and 12.58% after 17 months^1^. Fibular grafts showed a bone resorption rate of 5.30% after a mean observation time of 6 months, 8.26% after 11 months, and 16.95% after 17 months. Another study presented strong peri-implant bone resorption. However, there was no discernible difference between DCIA flaps and fibula flaps^44^.

The reduced success rate in the present study could have been due to the individual patient collective (large jaw defects), which is different from a patient with a single-tooth loss due to extensive jaw surgery. Implant success rates of 83.9% have been descirbed by Wiesli et al. in patients with microvascular fibula transplants^45^.

To avoid bias, an experienced clinician recorded all measurements. In addition, various factors can affect peri-implant health. Therefore, patients with uncontrolled diabetes, smoking habits and radiation therapy after dental implantation were excluded. It has been proven that heavy cigarette consumption has a negative effect on periodontal health^46^. Poor oral hygiene can also adversely affect the survival and success rate of implants.

The power with 1.00 is very high and may be interpreted as an over-confirmation of the study’s ability to detect an effect. The effect size was calculated based on a previous study^47^ and we used a sample size of 120.

In patients with reanastomized microvascular bony flaps, the literature describes problems such as peri-implant bone loss and inflammation of the soft tissues around implants inserted into different bone grafts or microvascular flaps^7,19^. An unfavorable crown–implant relationship, inflammation, and thick skin paddles are the current challenges in the treatment of patients with extensive microvascular jaw reconstructions^19,24^.

Future studies should focus on removable dentures with zirconia implants as patients with microvascular flaps are usually provided with removable prostheses. A short-term course of zirconia implants by means of an one-year study is important as due to the jaw reconstruction and the subsequent fixed tooth restoration the patient collective is not comparable with the standard implant case, of which there are many. Nevertheless, the authors believe that it is particularly important to examine these patients as well. As a disadvantage, however, it should be emphasized that the number of patients will rarely reach the same number. Further long-term studies of this collective are of great importance.

The strength of this study was to evaluate patients with fixed implant supported prothesis, as restorations in patients with jaw reconstructions are usually removable dentures. Future research should focus on a higher sample size and an extended follow-up period to improve the reliability and applicability of the findings. Additionally, a comparative group of titanium implants to strengthen the relevance and context of the findings should be included. To the best of our knowledge, there is no collective that has been treated with modern zirconia implants. Due to this highly individualized inclusion criterion, large sample sizes are scarce. Another limitation was the short observation period. Thus, the results of the present study must be interpreted with caution because of the small sample size.

## Conclusion

Regarding dental rehabilitation with zirconia implants and immunological parameters, no differences were found between flaps. Therefore, zirconia implants may be used in fibula and DCIA flaps for fixed dentures in the future; however, further studies with a higher number of patients and a longer observation period are necessary. Nevertheless, implant treatment with extensive microvascular jaw reconstructions affected the success rates in the present study (72.55% in the fibula group and 81.12% in the DCIA group).

## Data Availability

The datasets used and/or analysed during the current study are available from the corresponding author on reasonable request.
